# Complete Genome Sequence of a Tomato Isolate of Parietaria Mottle Virus from Italy

**DOI:** 10.1128/genomeA.01452-15

**Published:** 2015-12-17

**Authors:** Carolina Martínez, José Aramburu, Luis Rubio, Luis Galipienso

**Affiliations:** a Programa de Biología y Biotecnología Vegetal, Universidad Autónoma de Barcelona (UAB), Barcelona, Spain; b Independent Researcher, Barcelona, Spain; c Centro de Protección Vegetal y Biotecnología, Instituto Valenciano de Investigaciones Agrarias (IVIA), Valencia, Spain

## Abstract

We report here the complete genome sequence of isolate T32 of parietaria mottle virus (PMoV) infecting tomato plants in Turin, Italy, obtained by Sanger sequencing. T32 shares 90.48 to 96.69% nucleotide identity with other two PoMV isolates, CR8 and Pe1, respectively, whose complete genome sequences are available.

## GENOME ANNOUNCEMENT

Parietaria mottle virus (PMoV), a member of the genus *Ilarvirus*, family *Bromoviridae*, has a segmented positive single-stranded RNA (ssRNA) genome. PMoV was detected on tomato (*Solanum lycopirsicum*) and pepper (*Capsium annum*) plants showing mosaic and necrotic symptoms on leaves and fruits in some countries of the Mediterranean basin (Italy, France, Greece, and Spain). Limited nucleotide sequence data are available in GenBank, and only the complete genomes of two isolates have been sequenced: the Spanish isolate CR8, infecting tomato (1), and the Italian isolate Pe1, infecting *Parietaria officinalis* (2, 3). Here, we report the complete genome sequence of the PMoV isolate T32 obtained from a symptomatic tomato plant collected from a commercial orchard in Turin, Italy (4). Total RNAs were purified from infected plants using TRIzol reagent (Life Technologies, USA), denatured with 10 mM methylmercuric hydroxide, and polyadenylated by using yeast poly(A) polymerase (USB, USA) (5). After phenol-chloroform extraction and ethanol precipitation, the polyadenylated RNAs were reverse transcribed with SuperScript II reverse transcriptase (Life Technologies) with primer PM-1 (5′-CCGGATCCTCTAGAGCGGCCGC[dT]_17_V-3′), in which V represents A, C, or G, and then amplified with the Expand long-template PCR system (Roche, Switzerland), with a combination of PM-1 and primers designed based on Pe1 genomic sequences (1). The consensus nucleotide sequences of the genomic RNAs were obtained by sequencing overlapping PCR products of expected size in both senses with an ABI 3130XL genetic analyzer (Life Technologies). The genome of PMoV isolate T32 comprises three ssRNAs with 3,514, 2,921, and 2,268 nucleotides for RNA1, RNA2, and RNA3, respectively. The nucleotide identities of the T32 genomic RNAs were higher with the Pe1 RNAs (96.24, 94.01, and 96.69% for RNA1, RNA2, and RNA3, respectively) than with those of the CR8 (92.63, 90.48, and 93.78% for RNA1, RNA2, and RNA3, respectively). RNA1 encodes the putative polymerase P1 (1,097 amino acids), which shares 98.09% and 97.08% amino acid identities with Pe1 and CR8, respectively. RNA2 contains two overlapping open reading frames (ORFs) encoding the putative polymerase P2 and the putative silencing suppressor 2b. The amino acid identities were 96.40 and 93.30% with Pe1 and CR8, respectively, for P2 and 98.54 and 89.97% with Pe1 and CR8, respectively, for 2b. RNA3 encodes the movement protein (MP) and the coat protein (CP), separated by a noncoding intergenic region (IGR) (6). MP shared 97.95 and 96.59% amino acid identities with Pe1 and CR8, respectively, whereas CP shared an identity of 95.10% with Pe1 and 93.60% with CR8. The CP of T32 and CR8 had the same size but was 16 amino acids shorter than that of Pe1, as a consequence of a cytosine (C) deletion resulting in a different starting codon (1, 7).

In conclusion, this study determined the genome sequence of a new genotype of PMoV infecting tomato crops. T32 was collected in 1979 and is, to our knowledge, the first PMoV isolate reported.

### Nucleotide sequence accession numbers.

The nucleotide sequence of PMoV isolate T32 (RNA1, RNA2, and RNA3) was deposited in GenBank under the accession numbers KT005243, KT005244, and KT005245, respectively.
